# Supplementary material for: Dietary Barley Leaf Mitigates Tumorigenesis in Experimental Colitis-Associated Colorectal Cancer
Source: Nutrients. 2021 Sep 30;13(10):3487. doi: 10.3390/nu13103487 (PMC8537996; doi:10.3390/nu13103487)
Supplement: Supplementary file 1 [file nutrients-13-03487-s001.zip › nutrients-1394284-supplementary.pdf]

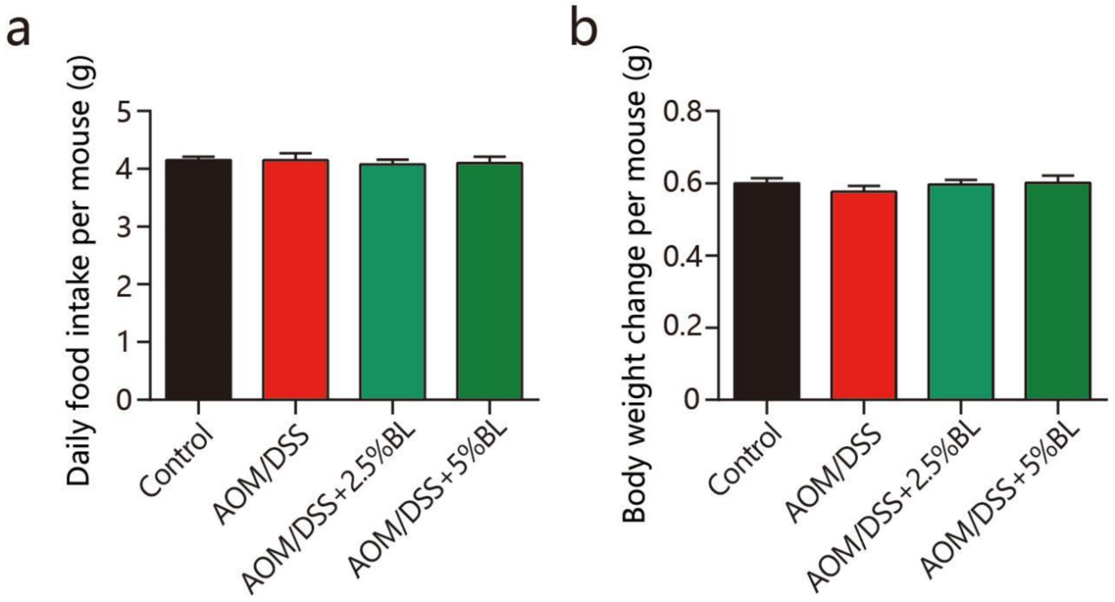

Supplementary Figure S1. Daily food intake and body weight change in the treatment groups. Mice were fed with a control diet or an isocaloric diet where BL was supplemented at a ratio of 2.5% or 5%. (**a**) Daily food intake and (**b**) body weight change were measured ($n = 10$). Data are mean ± SEM. One-way analysis of variance (ANOVA) followed by Tukey's multiple comparison's test. BL, barley leaf.

Supplementary Table S1. The macronutrient composition of the BL powder in the present study.

**Ingredients**	**Unit (g/100g)**
Protein	23.9
Fat	2.98
Carbohydrate	62.3
Soluble dietary fiber	<0.01
Insoluble dietary fiber	53.6
Moisture	3.99
Ash	6.8

Supplementary Table S2. The composition of control and BL-supplemented diet.

Ingredients (g/kg of Diet)	Diets		
	CD	2.5% BL	5% BL
Barley leaf powder	0	25	50
Casein	189.58	178.58	167.58
L-Cysteine	2.84	2.84	2.84
Corn Starch	298.59	280.59	262.59
Maltodextrin	33.18	33.18	33.18
Sucrose	331.77	327.77	323.77
Cellulose	47.40	47.40	47.40
Soybean oil	23.70	23.70	23.70
Lard	18.96	18.96	18.96
Mineral Mix M1002	9.48	9.48	9.48
DiCalcium Phosphate	12.32	12.32	12.32
Calcium Carbonate	5.21	5.21	5.21
Potassium Citrate	15.64	15.64	15.64
Vitamin mix V10001	9.48	9.48	9.48
Choline Bitartrate	1.90	1.90	1.90
Total	1000	1000	1000

All diets are isocaloric and contained 19.2% protein, 4.3% fat and 67.3% carbohydrate. CD, control; BL, barley leaf.

Supplementary Table S3. List of primers used in this study.

Genes	Primers	Primer Sequences (5′-3′)
iNOS	F	5′-CCCTTCCGAAGTTTCTGGCAGCAGC-3′
	R	5′-GGCTGTCAGAGAGCCTCGTGGCTTTGG-3′
COX-2	F	5′-GAAGTCTTTGGTCTGGTGCCT-3′
	R	5′-GCTCCTGCTTGAGTATGTCG-3′
ZO-1	F	5′-AGTTCTGCCCTCAGCTACCA-3′
	R	5′-GCTTAAAGCTGGCAGTGTC-3′
Occludin	F	5′-ACAAAGAGCTCTCTCGTCTCG-3′
	R	5′-CATAGTCTCCCACCATCCTC-3′
β-catenin	F	5′-TCTCCTTGGCTGGCCTTTCTA-3′
	R	5′-GTCACACAGCCCTGTCAAGA-3′
c-Myc	F	5′-GATGGAGATGAGCCCGACT-3′
	R	5′-CCTAGTGCTGCATGAGGAGAC-3′
Cyclin D1	F	5′-CTGCAAATGGAACTGCTTCTGGTGA-3′
	R	5′-AGCAGGAGAGGAAGTTGTTGGGGCT-3′
Axin2	F	5′-ACCAGGATGGTGCATACCTCT-3′
	R	5′-CCCATTACAAGCAAACCAGAAGT-3′
Lef1	F	5′-TGTTTATCCCATCACGGGTGG-3′
	R	5′-CATGGAAGTGTCGCCTGACAG-3′
TCF1	F	5′-AGCTTTCTCCACTCTACGAACA-3′
	R	5′-AATCCAGAGAGATCGGGGGT-3′
GAPDH	F	5′-GTGTTCCTACCCCCAATGTGT-3′
	R	5′-ATTGTCATACCAGGAAATGAGCTT-3
